# Mechanisms of Choice Behavior Shift Using Cue-approach Training

**DOI:** 10.3389/fpsyg.2016.00421

**Published:** 2016-03-23

**Authors:** Akram Bakkour, Christina Leuker, Ashleigh M. Hover, Nathan Giles, Russell A. Poldrack, Tom Schonberg

**Affiliations:** ^1^Imaging Research Center, The University of Texas at AustinAustin, TX, USA; ^2^Department of Psychology, Columbia University in the City of New YorkNew York, NY, USA; ^3^Center for Adaptive Rationality, Max Planck Institute for Human DevelopmentBerlin, Germany; ^4^Department of Psychology, Stanford UniversityStanford, CA, USA; ^5^Department of Neurobiology, Faculty of Life Sciences and Sagol School of Neuroscience, Tel Aviv UniversityTel Aviv, Israel

**Keywords:** cue-approach training, behavioral change, value-based decision making, attention, eyetracking

## Abstract

Cue-approach training has been shown to effectively shift choices for snack food items by associating a cued button-press motor response to particular food items. Furthermore, attention was biased toward previously cued items, even when the cued item is not chosen for real consumption during a choice phase. However, the exact mechanism by which preferences shift during cue-approach training is not entirely clear. In three experiments, we shed light on the possible underlying mechanisms at play during this novel paradigm: (1) Uncued, wholly predictable motor responses paired with particular food items were not sufficient to elicit a preference shift; (2) Cueing motor responses early – concurrently with food item onset – and thus eliminating the need for heightened top–down attention to the food stimulus in preparation for a motor response also eliminated the shift in food preferences. This finding reinforces our hypothesis that heightened attention at behaviorally relevant points in time is key to changing choice behavior in the cue-approach task; (3) Crucially, indicating choice using eye movements rather than manual button presses preserves the effect, thus demonstrating that the shift in preferences is not governed by a learned motor response but more likely via modulation of subjective value in higher associative regions, consistent with previous neuroimaging results. Cue-approach training drives attention at behaviorally relevant points in time to modulate the subjective value of individual items, providing a mechanism for behavior change that does not rely on external reinforcement and that holds great promise for developing real world behavioral interventions.

## Introduction

Monetary and food reinforcements have traditionally been employed to influence behavior (Thorndike, 1911; O’Doherty et al., 2004), but targeting automatic processes is likely more effective at attaining lasting behavioral change (Marteau et al., 2012). Previous research has established the cue-approach task as a reliable means to influence snack food choices for real consumption following a relatively short training period that does not employ external reinforcement or framing of the decision problem (Schonberg et al., 2014a). During cue-approach training, participants press a button on the keyboard in response to a neutral auditory tone that is consistently paired with approximately 25% of food stimuli. These “Go” food items that were paired with the tone and button press during training were later chosen for real consumption more often than other food items with equal pre-experimental preferences. Thus, after just an hour of training that involved no external reinforcements, we saw a shift in choice behavior. Several candidate mechanisms were put forth to account for this shift in preferences following non-reinforced cue-approach training, several of which we test in the current experiments: (1) cued attention alone modulates preferences (addressed in previous publication); (2) approach behavior alone modulates value for the trained action (Experiment 1); (3) internal reinforcement for correctly performing the training task modulates choice (Experiment 2); (4) cueing sustained top–down attention in anticipation of performing a motor approach response modulates item-specific subjective value (Experiment 3).

Development of the cue-approach task was largely inspired by the attentional boost effect (Lin et al., 2010; Swallow and Jiang, 2010), which refers to the counterintuitive finding that participants have better memory for images that were viewed concurrently with a behaviorally relevant target stimulus compared to images that were viewed concurrently with a distractor. The attentional boost effect is counterintuitive because most previous research described a memory deficit – rather than a benefit – for information learned under divided attention conditions (as is the case for the target condition in attentional boost paradigms, for review, see Mulligan, 2008). The cue-approach effect shares many commonalities with the attentional boost effect, but is distinct in several important ways. Most importantly, the effect is measured in value-based choice for cue-approach vs. episodic memory for attentional boost. Although episodic memories bias value-based decisions (for review, see Delgado and Dickerson, 2012; Shohamy and Turk-Browne, 2013; Palombo et al., 2015), the value of foods that govern choice in the cue-approach task are thought to be largely learned through non-declarative memory processes. The association between the food and tone cue during training does not fully explain choice behavior because participants do not choose those items any more than they choose non-associated items when foods are of relatively lower value in the stimulus set as is evident in studies 1 through 4 in Schonberg et al. (2014a). Thus the shift in preferences following cue-approach training is not explained solely by an attentional boost effect on memory for cue-associated foods. Furthermore, the attentional boost effect has typically been studied using rapid serial presentations of stimuli (non-word stimuli typically remained on the screen 100–500 ms). In contrast, during the cue-approach training task, food images remain on the screen for one second and trials are separated by an intertrial interval lasting between one and twelve seconds and averaging three seconds. These main differences, along with others discussed below, we believe make the cue-approach effect unique from the attentional boost effect. It is important to draw parallels between the two effects in terms of the importance of attention and behavioral relevance of attention orienting cues (Gottlieb et al., 2014), but to also appreciate the contribution of the cue-approach effect to understanding how values may be modulated to help more effectively change behavior. To better assist with the development of real-world behavioral change interventions based on cue-approach, we aim to better understand the cognitive mechanism by which preferences are modulated during training of the cue approach effect.

Development of the cue-approach training task was also heavily influenced by work on trained inhibition using the go/nogo or stop-signal training paradigms (for review, see Verbruggen and Logan, 2008b). In fact, the cue-approach task is the functional mirror of the cue-avoidance task in Studies 5 and 6 in Schonberg et al. (2014a). The cue-avoidance task we developed is highly similar to the ‘automated inhibition’ version of the stop-signal task (Verbruggen and Logan, 2008a; Lenartowicz et al., 2011). The cue-avoidance procedure was identical to cue-approach, except for the training phase. While during cue-approach training, participants responded with a key press only when they heard a tone cue, participants pressed a key on all trials *except* when they heard a tone cue in the cue-avoidance task. In our original published cue-avoidance studies and two additional unpublished studies, we did not see significant avoidance of stop-cue-associated food items during a choice phase identical to that used in the cue-approach studies. However, several other researchers have demonstrated a shift in preferences away from stop- or nogo-associated stimuli (Veling et al., 2013a,b; Houben and Jansen, 2015; Lawrence et al., 2015) or devaluation of stop-associated stimuli (Wessel et al., 2014), highlighting the potential of trained inhibition for development of real-world behavioral change paradigms. The cognitive mechanism underlying the shift in preferences following trained inhibition is under active investigation and the extensive literature that has thus far ensued offers some possibilities and some conundrums for understanding the mechanism underlying the cue-approach effect. In particular, recent work on the role of attention and expectancies in mediating response slowing to previously nogo-associated stimuli following modified go/nogo training revealed that stimulus-stop learning had a stronger effect on subsequent go performance when attention was higher to both task-relevant and task-irrelevant stimulus features (Best et al., 2015). In the present experiments, we focus on possible attentional mechanisms that may modulate the shift in preferences following cue-approach training.

Previous research has highlighted the importance of viewing time on choice preferences (Krajbich and Rangel, 2011). Additionally, manipulating visual attention during decisions influences choice behavior (Shimojo et al., 2003; Armel et al., 2008). Therefore, a simple mechanism of action during cue-approach training could be the modulation of preferences by attention captured when the auditory cue to particular items sounds. This mechanism does not rely on motor output, and eliminating the approach response should not affect the expected behavior change. However, in previous work, we showed that associating foods with a neutral tone without requiring a motor output did not result in a change in choice preferences (Schonberg et al., 2014a). This result is at odds with findings that the attentional boost effect does not require overt motor responses (Swallow and Jiang, 2012). More research on the parameters under which the cue-approach effect requires a motor response is necessary (e.g. covertly counting the cues without executing a motor response), however, we can rule out automated attention orienting due to an auditory cue on its own as a mechanism for modulating preferences in the cue-approach task.

Having shown that a motor response during cue-approach training is likely key to the shift in choice behavior, we posit that the combination of an auditory cue with a motoric approach response is necessary to induce the cue-approach choice effect. However, we have not tested the possibility that a motor response alone, paired with particular food items, is sufficient to measure a later change in choice preferences. In cue-approach studies, participants use the index finger of the right hand during the training phase, and then use the index and middle fingers to indicate left and right item choices, respectively, during the probe phase. In previous studies, we did not find a bias for left item choices (Schonberg et al., 2014a), suggesting that a simple stimulus-specific action (i.e., index finger–button press) association is not formed. This does not, however, preclude the possibility of a generalized approach behavior toward stimuli that had previously been associated with an approach response. To test the hypothesis that approach behavior alone modulates preferences, we eliminated the auditory cue to press a button and paired button presses with foods in blocks of trials in Experiment 1. Participants were instructed at the beginning of each block of trials to either press a button on every trial or to simply view items on the screen without pressing any buttons. In Experiment 1 presented here, maintaining attention on a trial-by-trial basis was not necessary, but we ensured that participants were viewing items equally between Go and NoGo blocks using eye-tracking (see exclusion criteria for Experiment 1 below). If approach behavior alone modulates preferences and attentional mechanisms are minimally at play, we would expect a shift in preferences following blocked training in Experiment 1. However, we believe that participants generate expectancies for the Go signal and subsequently increase top–down attention to Go items during standard cue-approach training. Thus, if attention plays an important role in the shift in preferences following cue-approach training, we expect that eliminating the need for participant-generated top–down attention during blocked training would eliminate the shift in preferences in Experiment 1.

An alternative mechanism responsible for a shift in choice behavior during cue-approach training is internal reinforcement for the subjective evaluation of correctly pressing a button when cued. To test this hypothesis, we presented the tone cue with the snack food item and instructed participants to press a button on the keyboard as fast as possible only when they heard an infrequent tone, but before the food item disappeared from the screen, a fixed second after onset in Experiment 2. Participants were told that they would not obtain feedback on button press successes, but that they would receive a small monetary bonus commensurate to their performance on the task and determined at the end of the experiment. We suspect that internal reinforcement, or the positive subjective feeling of having correctly pressed the button in time during training, does not rely on increased top–down attention. Thus, we presented the tone cue at the same time as the snack food appeared on the screen with no delay. In the standard version of the cue-approach task, the tone appears on average 750 ms after the onset of the food image on the screen. This go-signal-delay was titrated using a staircase procedure that ensured success (defined as pressing the button after the tone sounds, but before the image disappears from the screen a fixed one second after onset) on only 75% of all trials. Because the task in Experiment 2 is easier (since they have a full second rather than ∼250 ms to press a button), participants should have higher success rates. If the cue-approach effect relies on internal reinforcement for the subjective feeling of correctly pressing a button, we would expect a more dramatic shift in preferences following training in Experiment 2 than in the standard delayed cue design due to the higher success rate for pressing the button in time. However, our main hypothesis is that cue-approach training relies on heightened attention at behaviorally relevant points in time rather than internal reinforcement. We hypothesize that participants learn to expect a cue when a Go food item appears on the screen. We expect that heightened attention to detect the cue to perform an action modulates preferences. In Experiment 2, the tone sounds at the same time as the food image appears, thus no expectancies can be formed. Thus, we expect that eliminating the need for heightened attention to detect the cue in Experiment 2 yields no shift in preferences.

Whereas Experiments 1 and 2 address the relevance of behaviorally important cues and their timing to the cue-approach effect, neither address what type of values (values for the possible actions vs. intrinsic item-specific values) are being modulated during training. Decision-making in the cue-approach task involves choices between two food stimuli, each involving a different physical action (i.e., press a button with the index finger or press another button with the middle finger). Although we hypothesized that cue-approach training perturbs the value of stimuli directly, it remains possible that cue-approach training instead modulates values of the possible actions to indicate choice. If the latter is true, then the choice effect would be motor effector specific and we would see a shift in preferences when choices are executed using the trained motor effector (i.e., the finger), but not if the choice is executed using a different motor effector (e.g., eyes). The first indication that cue-approach training may not modulate action values lies in the fact that we found no bias for choosing the food item on the left of the screen using the index finger (the finger used to press the button when cued during training) in any of studies 1 though 4 in Schonberg et al. (2014a). We wanted to follow this observation up with a stronger test of our main hypothesis. We hypothesize that subjective value of individual items is modulated by heightened top–down attention to particular foods at behaviorally relevant points in time. We tested this hypothesis by training one motor effector (the hand) and tested choice using a different effector (the eyes) in Experiment 3. These two motor effectors were chosen because they each recruit distinct and dissociable networks of motor regions. The presence of two different networks for the hands vs. eyes can be used to test hypotheses about the motor responses required during the choice phase of the cue-approach task. It is possible that cue-approach training modulates value signals of possible actions at the supplemental motor area (SMA)/pre-SMA level (Wunderlich et al., 2009), but does not perturb the Go items’ intrinsic value at a higher level. In this case, cue-approach effects would not be present when participants choose using eye movements at the probe choice phase. Conversely, if value change in this task is achieved at a level independent of specific motor circuits, we should observe a standard cue-approach effect regardless of choice motor effector. To test this, in Experiment 3, participants were trained on a standard cue-approach training phase using their finger to press a button when they heard the cue tone that sounded after a variable delay following the onset of the food stimulus on the screen. In the probe choice for real consumption phase, participants were required to fixate on the item they would like to choose for 750 consecutive milliseconds to indicate their choice.

The set of experiments presented here test three main hypotheses and narrow the field of possible mechanisms responsible for the cue-approach effect. These findings help to better understand which automatic cognitive processes are targeted during the cue-approach task to achieve lasting behavioral change. The mechanism underlying the shift in preferences following cue-approach training is not yet fully understood. In the three experiments reported here, we address three questions: (1) Is a non-cued motor response sufficient to induce a shift in preferences? (2) Is the delay in cue appearance after the food stimulus onset required for a shift in preference? and (3) Is the shift in preferences motor effector specific?

## Materials and Methods

**FIGURE 1:**
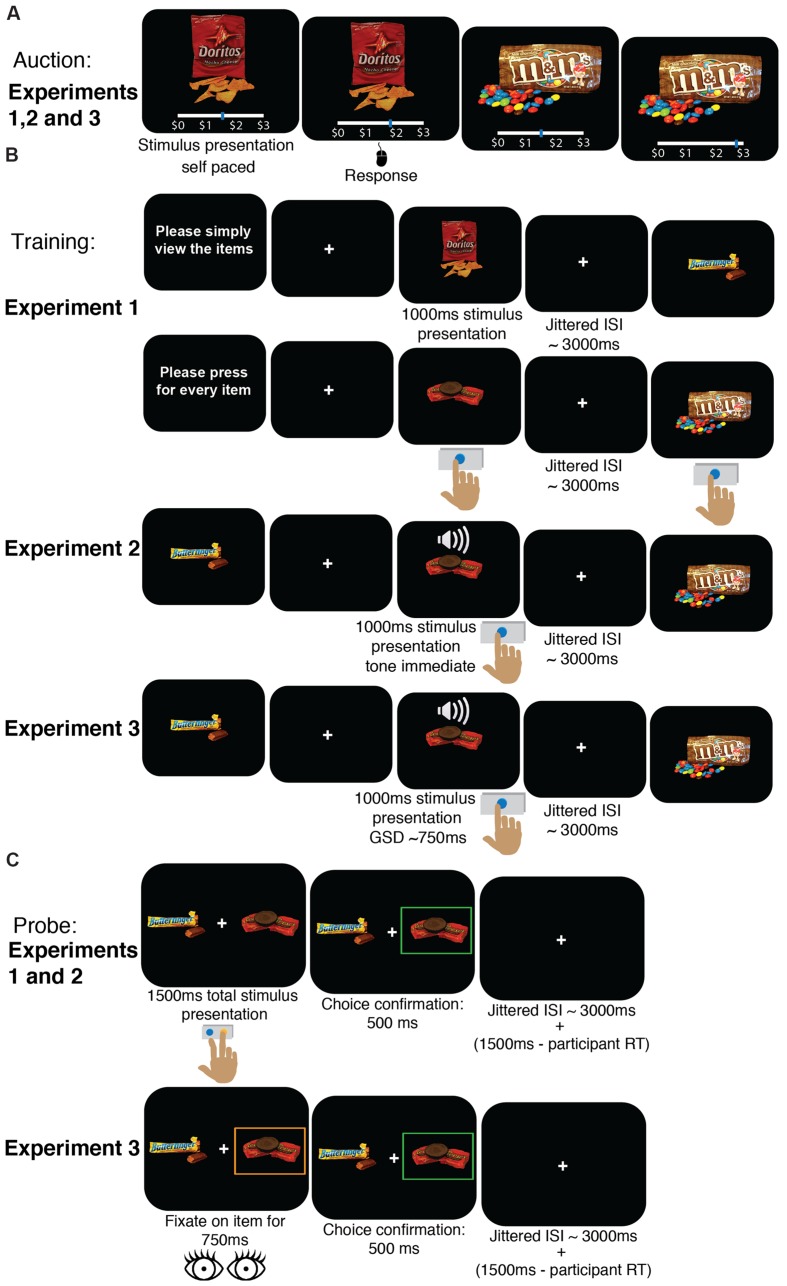
**Task procedure. (A)** Auction to obtain WTP for each item. This procedure was identical in all three experiments. **(B)** Training phase. In Experiment 1, participants were instructed at the beginning of each block to either press a button every time an item appeared on the screen or to simply view the items without pressing any buttons. In Experiment 2, participants were instructed to press a button when they heard a tone (that appeared concurrently with food onset, with no delay) but before the image disappeared from the screen (1 s after it appeared). In Experiment 3, training was standard, i.e., participants were instructed to press a button when they heard a tone (occurring after a variable delay based on a staircase) but before the image disappeared from the screen (1 s after it appeared). Images appeared on the screen one at a time, and ∼25% of items were associated with a tone. Trials were separated by a jittered intertrial interval (ITI) with a mean duration of 3 s. GSD, Go-signal delay. **(C)** Probe phase. Participants were instructed to choose one of two items that appeared on the screen to the right and left of a central fixation cross. Participants were told that a single trial would be selected and honored for real consumption, meaning they would receive the food item they chose on that particular trial to eat. Participants had 1.5 s to make their choice, and trials were separated by a variable intertrial interval with a mean duration of 3 s. In Experiments 1 and 2, participants made their choice using button presses, whereas in Experiment 3, participants were asked to fixate for 750 ms with their eyes on one of the items to indicate choice. RT, reaction time.

In the experiments reported here, we modified the standard cue-approach task to better understand the mechanisms responsible for a shift in preferences for appetitive junk food items. The standard cue-approach task implemented in studies 1 through 4 in Schonberg et al. (2014a) consisted of three phases: an auction (**Figure 1A**), a training phase (**Figure 1B**), and a probe phase (**Figure 1C**). For details of the procedures used, please refer to Schonberg et al. (2014a), but we will summarize them here then describe the differences in the procedure for each of the three new experiments.

### Stimuli and Procedure

Color photographs of 60 appetitive junk food items were used in this experiment. The same stimuli were used in previous experiments (Plassmann et al., 2007; Schonberg et al., 2014b,a). Stimulus presentation and behavioral data acquisition were implemented in python using Pygame (Shinners, 2011) for the auction, and in Matlab (Mathworks, Inc. Natick, MA, USA) using Psychtoolbox (Brainard, 1997; Kleiner et al., 2007) for the training and probe phases.

### Procedure for Standard Cue-approach Training

#### Auction

Upon arrival at the laboratory, participants were endowed with $3 and told that they would take part in an auction (**Figure 1A**). The auction followed the procedure outlined by Becker et al. (1964, BDM). Single pictures of food items appeared on the screen one at a time and participants placed their bid for each individual item by selecting a value on a visual analog scale at the bottom of the screen using a mouse. Participants were explicitly told that their best strategy for the auction was to bid exactly what the item was worth to them to buy from the experimenter at the end of the session. At the end of the experiment a single trial was selected at random and played out such that the computer generated a counter bid, which was a random number between 0 and 3 in 25 cent increments. This number was compared to the participant’s bid on the randomly selected trial and if the computer bid was higher than or equal to the participant’s, the participant could not buy that item. If, however, the computer bid was lower than the participant’s, then the latter was offered that item at the computer’s bid lower price. This auction provided us a measure of willingness-to-pay (WTP) for all 60 food items per participant.

#### Item Selection

We used WTP to rank order the foods for each participant from most preferred (highest WTP, rank order number 1) to least preferred (lowest WTP, rank order number 60). Items were split into high-value (rank order numbers 1–30) and low-value items (rank order numbers 31–60). Items were then placed into one of two training conditions; Go items required a button press during training and NoGo items required no response from the participant. Eight items were designated as Go items to later be paired with NoGo items matched for WTP in comparisons of interest during probe (see below): 4 high-value Go items (e.g., rank order numbers 8, 11, 12, and 15) each to be paired with 4 high-value NoGo items (e.g. rank order numbers 9, 10, 13, and 14) to yield sixteen unique high-value pairs, 4 low-value Go items (e.g., rank order numbers 46, 49, 50, and 53) each to be paired with 4 low-value NoGo items (e.g., rank order numbers 47, 48, 51, and 52) to yield sixteen low-value pairs of interest. This pairing procedure ensured that pairs of items presented during probe would be matched for WTP but differed on Go status, such that participants should a priori be indifferent in a choice between a Go and a NoGo item. To maintain ∼25% cue frequency as is common in stop-signal tasks (Logan and Cowan, 1984), we selected eight additional items to be paired with a Go cue during training: 4 high-value Go items (e.g., rank order numbers 16, 19, 20 and 23) to be paired with 4 low-value Go items (e.g., rank order numbers 38, 41, 42 and 45). These items will later be paired and used for high-value Go vs. low-value Go comparisons during probe. Full details of the pairing procedure can be found in Schonberg et al. (2014b).

#### Training

Participants viewed one food item at a time appear on the screen for 1 s followed by an inter trial interval (ITI) that lasted between 1 and 12 s and generated from an exponential distribution with mean 3 s (**Figure 1B**). Sixteen stimuli consistently required the participant make a button press on the keyboard (Go items, ∼25% of trials), while the rest (44 items) required no motor response (NoGo items). The order of Go and NoGo trials was randomized per block of 60 trials. Participants were told to press a button on the keyboard as quickly as possible only when they heard a tone. The auditory Go cue sounded a variable time averaging 750 ms after food stimulus onset and was adjusted using a 1-up/3-down staircase procedure that ensured that participants would successfully press the button in time on only 75% of Go trials. If the participant successfully pressed the button in time, go-signal-delay (GSD) was increased by 17 ms, making it harder to press the button in time on the next Go trial. If the participant failed to press the button in time after the tone, GSD was reduced by 50 ms, making it easier to press the button in time on the next Go trial.

#### Probe

After filling out a computer adapted version of the Barratt Impulsiveness Scale questionnaire (BIS-11, Patton et al., 1995) and on average 4 min after the end of training, participants were presented trials in which they chose between two items on the screen for real consumption (**Figure 1C**). On each trial, two food items appeared immediately to the right and left of a central fixation cross, and the participant was told to choose one item. They were told that a single trial would be selected at random at the end of the experiment and their choice on that trial would be honored for real, meaning they would receive the item they chose on that trial to eat at the end of the experiment. Each pair of interest was made up of two items with similar WTP; one was a Go and the other a NoGo item such that participants’ a priori preference for either item should be equal given their stated pre-experimental preferences measured by the auction. Full details of the pairing procedure are described in Supplementary Figure 1 of Schonberg et al. (2014a). Right–left item placement and pair presentation was randomized across trials and participants. Each of 32 unique pairs of interest and 32 unique pairs used for sanity checks (high- vs. low-value items) was presented twice for a total of 128 probe trials.

### Differences in Procedures for Current Experiments

#### Experiment 1

The auction was identical to the procedure described above. Training, however, was different. Participants viewed food items on the screen one at a time in blocks of Go or NoGo trials (**Figure 1B**, Experiment 1). At the beginning of each block participants were told to either press a button on the keyboard every time an item appears on the screen, but before it disappears in Go blocks or are told to passively view the items on the screen without pressing any buttons in NoGo blocks. Go items only appeared in Go blocks and NoGo items always appeared only in NoGo blocks. Stimuli appeared in random order per block. The order in which blocks appeared was counterbalanced across participants. Each of the 30 training items was repeated 15 times in different blocks for a total of 450 training trials. The probe task in Experiment 1 was identical to that described above; participants used the index and middle finger of their right hand to make choices (**Figure 1C**, Experiment 1).

#### Experiment 2

The auction was identical to the standard procedure described above. The training phase was very similar to the standard training procedure, but differed in the timing of the tone cue. Participants were instructed to view food stimuli appearing on the screen one at a time. They were instructed to press a button on the keyboard as quickly as possible and before the food stimulus disappeared from the screen only when they heard an auditory tone. In Experiment 2, the auditory cue always sounded immediately and concurrently with Go food stimuli presentation onsets (i.e., GSD = 0 ms, **Figure 1B**, Experiment 2). This contrasts with the cue-approach task in the original studies, in which the Go cue sounded after a variable delay (mean GSD = 750 ms) following the food stimulus onset. Each of the 60 items was presented 16 times for a total of 960 training trials. The probe task in Experiment 2 was identical to that described in Schonberg et al. (2014a); participants used the index and middle finger of their right hand to make choices.

#### Experiment 3

The auction was identical to the standard procedure. Participants also underwent standard cue-approach training identical to that described above. Each of the 60 items was presented 16 times for a total of 960 training trials. The probe phase was different from the standard procedure, however. It differed in the actions required to make a choice. Participants were required to make eye movements rather than manual button presses to indicate choice. Participants were asked to fixate on one of the two items on the screen for 750 ms continuously in order to confirm their choice for that item on each trial rather than press one of two buttons on the keyboard to indicate choice (**Figure 1C**, Experiment 3).

### Participants

Demographic details of the participant samples for the three experiments are described in **Table 1**. Briefly, Experiment 1 included 21 participants (15 female, mean age 21.2 ± 2.3), Experiment 2 included 25 participants (21 female, mean age 20.8 ± 2.3) and Experiment 3 included 25 participants (15 female, mean age 21.4 ± 2.8). Exclusion criteria are described below. Participants in the three experiments did not differ in age or BMI (*p*’s > 0.4). Sample sizes are similar to previously published studies (Schonberg et al., 2014b,a). All participants had normal or corrected-to-normal vision, no history of psychiatric, neurologic or metabolic illness, no history of eating disorders, no food restrictions and were not taking any medication that would interfere with the experiment. Participants were informed that the goal of the experiment was to study food preferences and were asked to refrain from eating or drinking anything besides water for four hours prior to their visit to the laboratory. All participants gave informed consent. The study was approved by the institutional review board (IRB) at the University of Texas at Austin.

**Table 1:** Participant demographic characteristics.

Expt	*N*	Gender (F/M)	Age		BMI		Excluded participants	Purpose of experiment
			*M*	*SD*	*M*	*SD*		
1	21	15/6	21.2	2.3	23.1	3.8	1 for auction 21 for viewing time	Effect of removing auditory cue
2	25	21/4	20.8	2.3	23.6	5.1	1 left handed	Effect of removing go-signal-delay
3	25	15/10	21.4	2.8	23.0	3.9	2 for auction 1 for training ladders	Effect of requiring a different motor effector during training and probe

*Participants who bid less than $0.25 on over 40 items during the auction were excluded. Participants who did not view the items as instructed during training were excluded. Participants whose training ladders (which govern the go-signal-delay) did not converge (indicating erratic behavior) were excluded.*

### Participant Exclusion Criteria

#### Auction Exclusion

Participants who consistently bid low on items during the initial auction did not provide us with enough range in bids to form pairs using the pairing procedure detailed in Schonberg et al. (2014a) and that matched items in pairs of foods to be used during the choice phase on stated subjective value. Thus we excluded one participant from Experiment 1 and two participants from Experiment 3 who bid less than 25 cents on 40 items or more during the initial auction.

#### Viewing Time Exclusion

Participants in Experiment 1 passively viewed items in blocks of NoGo trials and pressed a button on the keyboard every time a food appeared on the screen in blocks of Go items. Any observed shift in choice preferences that are due to differences in viewing time between Go and NoGo blocks would be explained by the mere exposure effect (Zajonc, 1968, 2001). We recorded participants’ gaze location on the screen using an infrared eyetracker during training in Experiment 1. The cue-approach effect is not explained by the mere exposure effect given that participants do not show differences in viewing time for Go vs. NoGo items during training in our original studies and that there were no differences in preference-related brain activation for Go vs. NoGo items at the end of training in our original imaging study (given that participants do not show differences in viewing time during training and that there were no differences in preference-related brain activation for Go vs. NoGo items at the end of training, Schonberg et al., 2014a). In order to eliminate the mere exposure effect as a potential explanation for any changes in preferences following modified cue-approach training in Experiment 1, we excluded 21 participants that viewed items during Go blocks (when they were pressing a button) more than when they were instructed to passively view items during NoGo blocks, but didn’t follow instructions and didn’t maintain their gaze on the food during NoGo blocks. Thus, the exclusion criterion was a significant difference in item viewing time (i.e. time spent fixating on the food) within subject for Go and NoGo blocks in Experiment 1. The unusually large number of excluded participants in Experiment 1 is due to the fact that this version is inherently different from the standard cue-approach task. In the standard set up, cue trials are presented randomly during training and thus participants need to maintain their vigilance to press the button on time before the items disappears from the screen. However, in Experiment 1, Go and NoGo items are presented in blocks and thus participants know they will not have to do anything during the NoGo block and potentially shifted their gaze and visual attention away from the images in NoGo blocks.

#### Training Ladder Exclusion

Participants in Experiment 3 underwent standard cue-approach training. The cue initially sounded 750 ms after the onset of a Go food on the screen. This GSD was adjusted on every Go trial using a staircase procedure. When participants pressed the button on time after the cue sounded, but before the image disappeared from the screen (a fixed one second after onset), GSD was increased by 17 ms on the next Go trial, making it more difficult to press the button on time. But if the participant failed to press the button in time, GSD was decreased by 50 ms on the next Go trial, making it easier to press the button on time. This 3:1 ratio ensured that participants would be accurate on about 75% of Go trials. Most participants’ GSD ladders converged around 750 ms. One participant in Experiment 3 was excluded from analysis because their ladders did not converge (i.e., GSD fluctuated throughout the training phase and did not asymptote as is typical), indicating that they were not following instructions and were behaving erratically during training.

### Eye Tracking

During training and probe in Experiments 1 and 3, we recorded participants’ eye movements using an Eyelink-1000 by SR Research (Mississauga, Ontario, Canada). We obtained coordinates for eye position on the computer screen at a rate of 250 Hz. Additionally, we used eye position data in real time in Experiment 3 by providing feedback to facilitate participants’ choices during the probe phase. Participants in Experiment 3 were required to fixate on one of the two items on the screen for 750 ms in order to confirm their choice of that item on each trial.

### Data Analysis

To test whether different forms of cue-approach training induced a preference change or whether using a different modality during choice reveals a preference shift, we performed repeated-measures logistic regression to compare the odds of choosing Go to NoGo items against equal odds for high-value and low-value pairs separately. To test any differences in reaction time (RT) or stimulus viewing time, we performed repeated-measures linear regression to compare these measures when participants chose Go vs. when they chose NoGo items.

## Results

### Experiment 1

We conducted this experiment to test the hypothesis that approach behavior alone modulates action values. To test this hypothesis, we eliminated the auditory cue and presented approach (Go) and no-approach (NoGo) item trials in blocks of trials. Each block was preceded by instructions indicating which block the participant was about to start.

#### Choice

**FIGURE 2:**
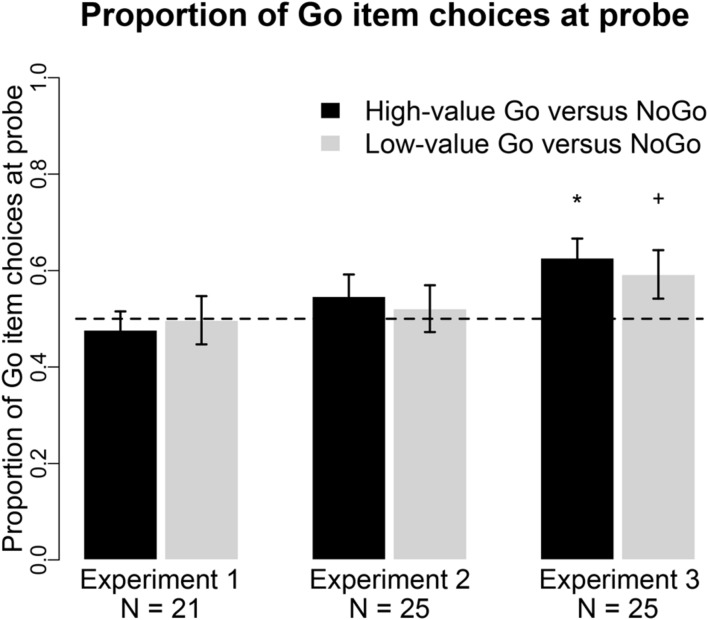
**Probe choice.** Proportion of choices of the Go item in pairs of high-value Go vs. NoGo (black bars) and low-value Go vs. NoGo items (gray bars) in all three experiments. *p < 0.01, ^+^*p* < 0.05 in two-tailed repeated measures logistic regression.

Eliminating the auditory cue to press a button during cue-approach training, and thus rendering button presses completely predictable, eliminated the shift in preferences toward Go items. **Figure 2** summarizes the probe behavioral results in Experiment 1. Participants chose Go over NoGo items on 48% of high-value pair trials [odds ratio = 0.90, 95% CI = [0.65 1.24], *p* = 0.5 for odds of choosing high-value Go to NoGo items, Bayes Factor in favor of the null (BFn) = 5.06] and 50% of low-value trials (odds ratio = 0.97, 95% CI = [0.62 1.52], *p* = 0.9 for odds of choosing low-value Go to NoGo items, BFn = 10.15). The high-value pair choice effect in Experiment 1 are significantly different than the effect in the four original studies that employed the standard cue-approach design in Schonberg et al. (2014a), odds ratio = 2.05, 95% CI = [1.34 3.15], *p* = 0.001 for choices of high-value Go items in Experiment 1 compared to the four original studies). The low-value pair effect in Experiment 1 did not differ from the effect in the four original studies (odds ratio = 1.32, 95% CI = [0.78 2.23], *p* = 0.3, BFn = 3 for choices of low-value Go items in Experiment 1 compared to the four original studies).

#### Eyetracking

**FIGURE 3:**
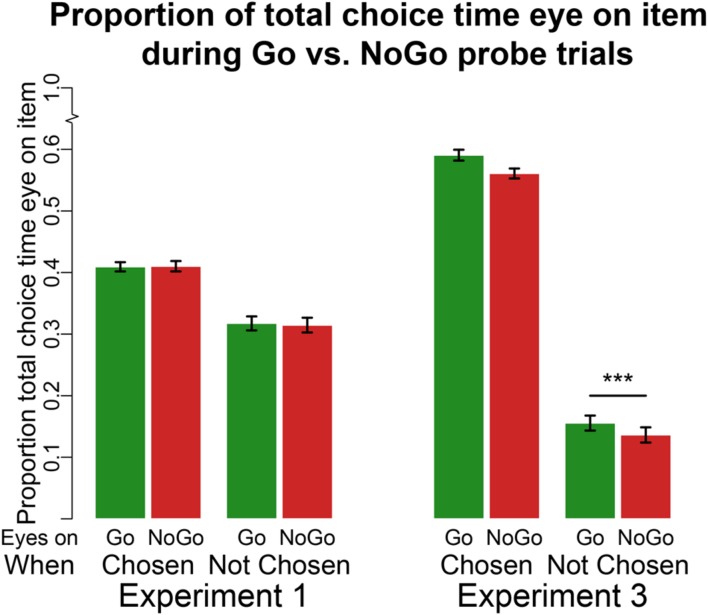
**Eyetracking at probe.** Proportion of choice time eyes on Go (left green bar in each pair of bars) or NoGo (right red bar in each pair of bars) item either when that item is chosen (set of two bars on the left) or not chosen (set of two bars on the right) for Experiments 1 (four bars on the left) and 3 (four bars on the right). ***p < 0.0001 in two-tailed repeated measures linear regression.

In line with previous findings (Shimojo et al., 2003; Armel et al., 2008; Schonberg et al., 2014a), there was a main effect for chosen items (regardless of Go/NoGo status) on the proportion of choice time spent viewing an item (**Figure 3**, mean proportion for chosen item = 0.41, mean proportion for unchosen item = 0.32, β = 0.09, 95% CI = [0.08 0.10], *p* < 0.0001). However, unlike previous findings using the standard cue-approach task, there was no main effect of Go status on the proportion of time participants viewed the item (mean proportion of choice time viewing Go items = 0.36, NoGo = 0.36, β = 0.002, 95% CI = [–0.01 0.01], *p* = 0.8, BFn = 22.02). There was no interaction between item chosen/unchosen and Go/NoGo status on proportion of time spent viewing the item. These results suggest that training with no auditory cue did not bias attention toward Go items. Previous findings showed that participants tended to look at the Go item longer, even when that Go item was not chosen (Schonberg et al., 2014a).

#### Reaction Time

Participants were on average slower at choosing between low-value items than they were choosing between high-value pair items (low-value choice mean RT = 871.4 ms, high-value choice mean RT = 839.1 ms, β = 33.75, 95% CI = [13.1 54.4], *p* = 0.001). There was no interaction between pair type (high- or low-value pairs) and choice of Go or NoGo on RT (β = 8.9, 95% CI = [–34.2 52.2], *p* = 0.7, BFn = 14.03). RT also did not differ for choices of Go or NoGo (β = 5.95, 95% CI = [–15.7 27.6], *p* = 0.6, BFn = 14.21).

### Experiment 2

We conducted Experiment 2 to test the hypothesis that internal reinforcement for correctly performing the training task modulates choice. This hypothesis posits that vigilance, or heightened top–down attention is not required during the cue-approach training. We test this hypothesis by eliminating the Go signal delay (GSD) – i.e., the delay to sound the auditory cue to press a button after food stimulus onset – during cue-approach training.

#### Choice

Eliminating the delay between food stimulus onset and auditory cue onset during cue-approach training eliminated the shift in preferences toward Go items. **Figure 2** summarizes the probe behavioral results in Experiment 2. Participants chose Go over NoGo items on 55% of high-value pair trials (odds ratio = 1.27, 95% CI = [0.83 1.93], *p* = 0.3 for odds of choosing high-value Go to NoGo items, BFn = 0.39) and 52% of low-value trials (odds ratio = 1.08, 95% CI = [0.69 1.71], *p* = 0.7 for odds of choosing low-value Go to NoGo items, BFn = 5.66). The choice effect for the high-value pairs was marginally lower than in the four previous studies in Schonberg et al. (2014a), odds ratio = 1.47, 95% CI = [0.96 2.25], *p* = 0.08, for choices of high-value Go items in Experiment 2 compared to the four original studies). The choice effect in the low-value pairs did not differ from the effect in the previous samples (odds ratio = 1.17, 95% CI = [0.71 1.93], *p* = 0.5, BFn = 3.84 for choices of low-value Go items in Experiment 2 compared to the four original studies). These choice effects do not differ between Experiments 1 and 2 (*p*’s > 0.2, BFn’s > 1.89).

#### Reaction Time

Reaction times did not differ between low-value and high-value pair choices. RTs were also the same for choices of Go and NoGo items (all *p*’s > 0.2, β’s < 12.5, and BFn’s > 7).

### Experiment 3

We conducted Experiment 3 to test the hypothesis that cueing sustained top–down attention in anticipation of performing a motor approach response modulates item-specific subjective value. If item-specific values rather than action values are being modulated, the choice effect should not be motor effector specific. To test this hypothesis, participants were trained using manual button presses but were asked to indicate choice during probe using eye movements.

#### Choice

Using a different motor effector (eye rather than hand) during the probe phase revealed a choice preference for Go items following standard cue-approach training. Cue-approach training likely affects item valuation/processing rather than simpler action values. **Figure 2** summarizes the probe results in Experiment 3. Participants chose Go over NoGo items using eye movements on 63% of high-value pair trials (odds ratio = 1.83, 95% CI = [1.25 2.68], *p* = 0.002 for odds of choosing high-value Go to NoGo items) and 59% of low-value trials (odds ratio = 1.59, 95% CI = [1.04 2.42], *p* = 0.03 for odds of choosing low-value Go to NoGo items). The choice effects in Experiment 3 did not differ from those in the previous four studies in Schonberg et al. (2014a), *p*’s > 0.4, BFn’s > 2.93). Choices of high-value Go items were significantly higher in Experiment 3 when compared to choices in Experiment 1 (odds ratio = 2.02, 95% CI = [1.22 3.35], *p* = 0.006 for choices of high-value Go items more prevalent in Experiment 3 compared to Experiment 1), but not significantly different than in Experiment 2 (odds ratio = 1.46, 95% CI = [0.83 2.57], *p* = 0.2, BFn = 1.87 for choices of high-value Go items in Experiment 3 compared to Experiment 2). Choices of low-value Go items were not different between any of the experiments (odds ratios < 1.64, *p*’s > 0.1, BFn’s > 1.43).

#### Eyetracking

Participants were instructed to fixate on the item they would like to choose for 750 ms in order to execute their choice. Thus, the main effect of choice on the proportion of choice time the eyes are fixated on a particular item is artificial (see **Figure 3**). However, we ran a mixed effects linear regression model examining the effect of Go status (two levels: Go and NoGo) on proportion of choice time viewing a particular food with participant as a grouping factor. We found a main effect of Go status on viewing time (mean proportion of choice time viewing Go items = 0.42, NoGo = 0.30, β = 0.12, 95% CI = [0.10 0.12], *p* < 0.0001). This finding replicates previous results using eyetracking during the standard cue-approach task when participants chose between the two items using button presses (i.e. using the same motor effector that was trained). Moreover, using the same mixed-effects model above on data for times participants were fixated on unchosen items only, we found a simple effect of Go status on viewing time within unchosen items (mean proportion of time spent viewing unchosen Go = 0.16 and viewing unchosen NoGo = 0.14, β = 0.02, 95% CI = [0.01 0.04], *p* < 0.0001). Participants viewed Go items longer than they viewed NoGo items even when the item was not ultimately chosen.

## Discussion

We have recently shown that choices can be influenced using the novel cue-approach paradigm that does not rely on external reinforcement or re-framing of the decision problem (Schonberg et al., 2014a). The findings of the experiments described here shed light on the mechanism by which preferences shift during cue-approach training. This was achieved by manipulating several aspects of the basic cue-approach task design. In Experiment 1, we eliminated the tone that cues participants to perform a motor action, instead presented food items in blocks of trials and instructed participants to passively view items or to press a button every time a food appears on the screen. In this experiment, we found no evidence of a shift in choice preferences following blocked training, consistent with our view that motor approach alone is not sufficient to elicit a change in preferences. In Experiment 2, we eliminated the delay between the onset of the food image and the tone cue to press a button. This made the task easier for participants to perform and did not allow for anticipation of the tone when a Go food item appeared on the screen. We found no evidence of a change in preferences following modified training that omitted the delay, consistent with our hypothesis that top–down attention directed at the foods during anticipation of the tone is key to a shift in preferences. Finally, in Experiment 3, we required a different motor effector during standard cue-approach training and choice phases. When participants used eye movements to make choices, we found evidence of a significant shift in preferences on par with findings in our original studies where participants used their fingers during both training and choice phases.

Cueing a motor response during training appears to be important for the shift in choice preference. In the standard cue-approach training task, the food stimulus is presented first, followed by the cue to perform a motor response. Performing an uncued motor response during blocks of training trials at the beginning of which the participant receives instructions to follow for the whole block of trials (press a button or passively view items), does not lead to a shift in choice behavior (**Figure 2**, Experiment 1). This finding, in combination with our previous finding that an auditory cue in the absence of a motor response is also not sufficient to induce a change in choice preferences, reinforces our claim that attentional as well as motor mechanisms are likely at play during cue-approach training. It should be noted that the design of this version is also different from the original task as the presses are entirely predictable and do not involve heightened anticipation. Due to this fact we also incurred a very high percentage of excluded participants as they were not watching the items that were not associated with a button press. However, in a post hoc analysis of the probe phase data including all participants did not change the pattern of results. Excluding participants that did not view the Go and NoGo items equally ensured that mere exposure was not a factor in this experiment and that participants were indeed maintaining visual attention on the foods equally in both task conditions. An alternative task design was considered where participants are instructed to press the button when they wanted to without a cue as was implemented in Swallow et al. (2012). However, this design would have required only a single participant-determined button press-food pairing whereas food-cue-button press pairings were repeated (8, 12, or 16 times per item) in previous standard cue-approach training phases. The block design in Experiment 1 here also allowed us to maintain the same controlled food pairing procedure that matched values in choice pairs of items based on the initial auction used in previous standard versions of the task. The absence of an auditory cue in the version of the cue-approach task implemented in Experiment 1 eliminates the need for focusing attention at behaviorally relevant points in time. The absence of a need for sustained top–down attention usually initiated by an expectation of the forthcoming cue to make a motor response once the block of training trials commences, despite maintenance of visual fixation on the foods, is likely responsible for the lack of a behavioral or eyetracking effects in Experiment 1. These findings are consistent with the view that an auditory cue along with a motor response during training are essential to elicit a shift in preferences in this task. However, future research should investigate different types of cues that may affect cue-approach training differentially. To date, only neutral tones have been employed as the cue to perform a motor action. Additionally, although Experiment 1 results are consistent with the view that an approach response alone is not sufficient to lead to a change in preferences, the response in this version of the cue-approach task is instructed. Perhaps agency is important for the inherently valenced approach response to have an effect on value of foods. Future research should employ a task design similar to the alternative design described above to test this possibility and provide fuller understanding of the contributions of motor and attentional mechanisms in the cue-approach task.

External reinforcement on a trial-by-trial basis has been shown to be effective at influencing behavior (Thorndike, 1911; O’Doherty et al., 2004; Tricomi et al., 2009; Schonberg et al., 2014b). However, the effectiveness of this strategy on long-term behavioral change has been questioned (Marteau et al., 2012). Thus, we were inspired to develop a paradigm that did not rely on external reinforcement and showed that cue-approach training had an effect on preferences that lasted longer than a month (Schonberg et al., 2014a). We could not however control participants’ subjective feelings during the training task and wanted to test the possibility that internal reinforcement for correctly pressing the button when cued was responsible for a shift in preferences. This form of reinforcement would presumably be equally vulnerable on the long-term as external reinforcement. In Experiment 2, we eliminated the delay between onset of the food image on the screen and the sounding of the tone cue. In this version of the task, participants achieved a higher rate of success than in the standard version as they had more time to press the button in time after the cue sounded, thus would presumably receive more internal reinforcement. If internal reinforcement played a role during cue-approach training, we would expect a larger effect on preferences at the choice phase in Experiment 2. We found no evidence of change in preferences following training that yielded more correct responses, suggesting no role for internal reinforcement. This version of the task however is significantly easier for the participants to perform than the standard version, given that the tone sounds concurrently with the food stimulus onset in Experiment 2. The original studies employ a staircase procedure that ensures that participants are successful at pressing the button after the tone sounds and before the food image disappears from the screen on only three quarters of all trials. Given the discrepancy in task difficulty, a limitation of the design in Experiment 2 is the possibility that participants do not receive as much internal reinforcement in this easier task compared to the standard design. Future studies should measure the subjective value of being correct in this task to ascertain its role in modulating food value during cue-approach training.

Attention has been shown to significantly modulate value. When participants view items longer they tend to later choose them (Krajbich and Rangel, 2011) and experimentally biasing visual attention influences choice (Shimojo et al., 2003; Armel et al., 2008). However, in the standard cue-approach task, viewing times for the Go and NoGo items did not differ during the training phase (Schonberg et al., 2014a). This suggests that mere exposure did not play a significant role in this task and cannot account for the choice phase findings. Automated attention capture on its own also does not appear to be sufficient to induce a shift in preference since a tone cue that does not require a motor response does not lead to a bias in choice for cued items (Schonberg et al., 2014a). It remains unknown, however, whether requiring a covert task such as counting without an overt motor response would lead to a shift in choice preferences. However, attention clearly plays a significant role in this task. Eliminating the delay between the onset of food stimulus and the auditory cue to press a button during the training phase in Experiment 2 weakened the choice effect at the later probe phase (**Figure 2**, Experiment 2). Choices for Go over NoGo items were not significant, but were only marginally lower than in previous studies. This suggests that sustained attention toward particular Go items enhances the modulation of preferences during the cue-approach task. After participants learn to anticipate the tone once a Go item appears on the screen in the standard cue-approach design, they focus more intently on that item in anticipation of the cued motor response. However, the tone onset time in the standard version of the task is not perfectly predictable since it sounds at a variable time after the food stimulus onset to ensure 75% Go success. The timing of the cue during the cue-approach task seems to play a more central role than in the attentional boost task, where overlap in time between the to-be-remembered image and the target is crucial, but the timing of the overlap has been shown to matter little (Swallow and Jiang, 2011). Further research is needed to elucidate whether the uncertainty in the timing of the cue is key to the cue-approach choice effect, or if anticipation of the cue, even if onset time is perfectly predictable, is sufficient. By eliminating GSD, we reduced the time during which top–down attention is potentially sustained toward Go items before a behavioral response is executed. Eliminating the need for sustained attention has potentially reduced its modulatory effect on the value of Go items. Not only is a cued motor response apparently necessary for the cue-approach effect, but also the cue must appear some time after the food stimulus onset. The findings from Experiment 1 and 2, however, do not preclude the possibility that lower-level attentional mechanisms rather than higher-level top–down attention is engaged during cue-approach training. This possibility could be resolved by future research examining the explicit awareness of participants for the food-Go contingencies. We have some unpublished data from a recognition memory test that suggests that participants were aware of these contingencies, but better tests of explicit awareness are needed for more conclusive evidence. We suspect that greater awareness of the contingencies will lead to greater shifts in preferences following cue-approach training, analogous to findings by Wessel et al. (2015) that show greater stimulus devaluation following a stop-signal task when the value representation for those stimuli is explicit.

Experiments 1 and 2 yielded expected null results consistent with our main hypothesis, suggesting that approach responses along with an expectancy for the cue may play important roles in modulating value of foods during cue-approach training. However, the nature of the values modulated in this task remained untested. It remained possible that during cue-approach training, value for the action (pressing a button with the index finger) rather than item-specific intrinsic value was being modulated. If action value was being modulated and played a role in the cue-approach effect, we would expect there to be a bias toward choices executed using the index finger (which is the trained effector). However, we found no bias toward choices made with the index finger in our previous studies employing standard cue-approach training (Schonberg et al., 2014a). Furthermore, in Experiment 3 here, participants used a different motor effector (eyes) than the trained motor effector (finger) to make choices. Although non-saccadic decisions remain dependent on the visual network when stimuli are presented visually, the actual motor responses in value-based decisions made with the eyes vs. the hands recruit dissociable motor networks. Participants in Experiment 3 exhibited a choice bias in favor of Go items previously associated with a cued manual button press during the training phase. These findings suggest that intrinsic item-specific value rather than action value is being modulated during the training phase to lead to a choice preference at the choice phase. Requiring participants to choose between two items that were equated for pre-experimental preferences but differed on Go status using eye movements rather than button presses (i.e., a different motor effector than the trained effector) did not eliminate the Go choice effect. Given these findings, cued button presses seem to focus attention at behaviorally relevant points in time during cue-approach training, which likely modulates intrinsic item value rather than the value assigned to the action of pressing a button with the index finger. However, more research on the nature of values modulated during cue-approach is warranted. Although we did not find an effect on value as measured by a second auction *in lieu* of binary choice following standard cue-approach training in Study 9 in Schonberg et al. (2014a), we were likely underpowered to detect a subtle effect in that study, especially considering that the measurement of willingness-to-pay is susceptible to regression-to-the-mean during the second auction. Future studies that employ a method of measuring item-specific value that does not rely on binary choice or a BDM auction could shed more light on the nature of the values modulated during non-reinforced training in the cue-approach task.

## Conclusion

Further evidence is consistent with our hypothesis that the cue-approach task works at the level of modulating individual items’ intrinsic value by driving attention toward those items at behaviorally relevant points in time. Thus far, we have only shown that the value of initially already high-value stimuli (appetitive snack foods) can be boosted following cue-approach training. For compelling relevance of the cue-approach task for real-world applications, future work should investigate the effectiveness of this training in shifting preferences toward initially lower-valued stimuli such as less palatable but healthier foods, for example. However, this research has already modestly improved our understanding of how value can be modulated and holds great promise in the development of novel real-world behavioral change interventions.

## Author Contributions

AB, RP, and TS designed the studies. AB, AH, and NG programmed the tasks. CL, AH, and NG collected the data. AB, CL, and TS analyzed the data. AB, RP, and TS wrote the paper.

## Conflict of Interest Statement

The authors declare that the research was conducted in the absence of any commercial or financial relationships that could be construed as a potential conflict of interest.
